# Subjective assessment and biochemical evaluation of traction therapy in women with chronic low back pain: does body mass index matter? A clinical study

**DOI:** 10.1186/s12891-023-06300-5

**Published:** 2023-03-16

**Authors:** Marzena Ratajczak, Michał Wendt, Ewa Śliwicka, Damian Skrypnik, Jacek Zieliński, Krzysztof Kusy, Piotr Krutki, Małgorzata Waszak

**Affiliations:** 1 Department of Biology and Anatomy, Poznan University of Physical Education, Królowej Jadwigi 27/39, 61-871 Poznan, Poland; 2 Department of Physiology and Biochemistry, Poznan University of Physical Education, 61-871 Poznan, Poland; 3 Department of Treatment of Obesity, Metabolic Disorders and Clinical Dietetics, Poznan University of Medical Sciences, 61-701 Poznan, Poland; 4 Department of Athletics, Strength and Conditioning, Poznan University of Physical Education, 61-871 Poznan, Poland

**Keywords:** Lumbar traction, Visual analogue scale, Pressure pain threshold, CS-846, Neuropeptide Y, GDF-15, Leptin, Adipsin, Lower back pain, Traction forces

## Abstract

**Background:**

Apart from the positive effect of lumbar traction on structural changes within the spine in patients with low back pain, it is likely that therapeutic effects are correlated with pain biomarkers in the blood. Among them, systemic metabolic factors related to obesity may play an important role. This is the first study designed to examine the effectiveness of traction therapy in two experimental groups with considerably different BMI and to assess relationships between blood biomarkers and low back pain intensity.

**Methods:**

In the prospective clinical trial, women suffering from chronic low back pain were allocated into the normal-weight or obesity groups. Patients in both groups underwent twenty sessions of lumbar traction therapy (30 min a day, continuous mode with a force level of 25–30% of body weight). Before and after therapy subjective assessments of pain (VAS and PPT) were performed, and serum concentrations of aggrecan chondroitin sulfate 846 epitope (CS-846), neuropeptide Y, leptin, adipsin and growth and differentiation factor 15 (GDF-15) were determined. The data were statistically evaluated for 28 women.

**Results:**

After therapy, the maximal low back pain decreased in both groups, GDF-15 concentration was reduced in the normal-weight group and increased in the obesity group, and CS-846 concentration decreased in the obesity group. The sensation of PPT in the lumbar spine and mean concentrations of neuropeptide Y, leptin and adipsin did not change in both groups. However, the relationships of GDF-15, leptin, and adipsin concentrations with the perception of pain were revealed.

**Conclusion:**

Distinct differences between the normal-weight and obesity groups pointed on the role of excessive adipose tissue in aggravating the inflammatory processes and in the development of low back pain. Adipsin, CS-846 and GDF-15 aspire to be the low back pain biomarkers in women with obesity, but there is a need for further research to answer whether they might be considered reliable biomarkers for the prognosis and monitoring of chronic low back treatment.

**Trial registration:**

NCT04507074, registered prospectively on July 6, 2020.

## Introduction

Systemic biomarkers which correlate with low back pain, a function related to pain or degeneration of the spine, have recently received a lot of attention. Indicators are being sought to predict disease progression and monitor treatment, and to serve as diagnostic tools as well as provide insight into both the etiology of back pain and the mechanisms of therapy.

A large amount of literature data confirms that the presence of structural damage within the spine can be measured in the peripheral blood [1]. There are also reports showing a relationship between the concentration of substances circulating in the blood and pain symptoms in the lumbar spine and the functioning of the patient [2–5]. The influence of several types of interventions on changes in the concentration of biomarkers correlating with back pain has also been demonstrated [5–7].

The application of traction forces has beneficial effects in degenerated intervertebral discs [8]. Animal studies confirm that spine stretching manifests itself in the regeneration of anatomical structures and improved hydration of the discs [9]. The influence of traction therapy on the concentration of blood components, pretending to be markers of low back pain, has not been investigated so far. However, the positive effect of traction therapy on structural changes suggests that its therapeutic effects may occur in the blood of patients as well. Traction forces result in stretching the tissues within the spine, which reduces the pressure on damaged intervertebral discs and disc decompression may alleviate the chronic inflammation in the affected area causing favorable biochemical changes in the blood of the examined patients.

Due to the fact that the content and distribution of adipose tissue is associated with the intensity of lower back pain and the related disability [10], it is assumed that systemic metabolic factors related to obesity also play an important role in the pathogenesis of low back pain. Recent studies suggest that two key processes, sarcopenia and dysregulation of adipose tissue activity are important in the development of lower back pain [4] and the degree of this infiltration correlates with the perception of pain [11]. Fatty acids and their derivatives accumulate in the muscles and induce inflammation [12]. Lipotoxicity, together with impaired mitochondrial function and chronic inflammation, may be one of the mechanisms leading to the degeneration of intervertebral discs, sarcopenia, and the development of chronic low back pain. It has been hypothesized that this vicious circle mechanism, in which biochemical and functional changes manifested by back pain exacerbate sarcopenia and inflammation, may be stimulated by a factor inhibiting the growth of muscle and bone tissue, e.g. growth and differentiation factor 15 [4]. Regardless of the trigger factor, sarcopenia, obesity or sarcopenic obesity, consequently leading to excessive secretion of fatty acids and adipokines, contribute to the generally detrimental environment that favors the development of low back pain. There are also premises that concentrations of cytokines and adipokines differentiate individuals with back pain from individuals without back pain [13].

The first goal of this study was to examine the effectiveness of traction therapy in two experimental groups (women with normal body weight and women with obesity) suffering from chronic pain in the lumbar spine, in relation to the intensity of pain, sensitivity to touch and changes in the concentration of biochemical components in the blood. This is a novelty, because so far, studies on biomarkers correlating with back pain have been carried out in the population of people with back pain regardless differences in body weight [2, 5, 14]. Such a design will allow us for an insight into the likely different etiology of back pain in slim and obese people, but also to observe possible different reactions of the body to the intervention in the form of the introduction of traction forces.

The second goal of the study was to assess relationships between biomarkers and pain intensity and sensitivity to touch in the lumbar spine. This is the first attempt to answer the question whether traction therapy can cause changes in the potential biomarkers of back pain in the blood? On a basis of the latest literature, for the evaluation of biochemical changes induced by traction therapy we selected the aggrecan chondroitin sulfate 846 epitope (CS-846), the pain-related neuropeptide-Y and three molecular biomarkers related to adipose and muscle tissue metabolism: leptin, adipsin, and the growth and differentiation factor 15 (GDF-15). So far, the relationships between the above-mentioned markers and the intensity of pain or the pressure pain threshold have been unknown and it is uncertain whether they reflect an active disease process leading to back pain.

The following hypotheses were drawn: [1] the application of traction forces in the subjects, through decompression of damaged and chronically inflamed intervertebral discs, will result in favorable biochemical changes in the blood and alleviation of pain, confirmed by a reduction in the threshold and intensity of pain in the lumbar region; [2] the effects of the therapy will be more noticeable in the group of women with normal body weight.

## Methods

### Study design

Study aimed to examine the effectiveness of traction therapy in two experimental groups with considerably different BMI and to assess relationships between blood biomarkers and low back pain intensity. The study was designed as a prospective clinical trial with two experimental groups and adhered to the standards laid down in the Declaration of Helsinki. The study research protocol was approved by the Ethics Committee at the Poznan University of Medical Sciences in Poland (ref. 958/19). The study protocol was registered in the ClinicalTrials.gov database (NCT04507074) and can be found at: https://clinicaltrials.gov/ct2/show/NCT04507074?recrs=d&cntry=PL&city=Pozna%C5%84&draw=2&rank=3. Women suffered from the chronic low back pain were allocated into two groups: normal-weight women and women with obesity. Both groups underwent twenty sessions of lumbar traction therapy. Aside from the therapy, all patients were instructed to maintain their normal physical activity and diet which they have been practicing so far. At baseline and after twenty sessions of the therapy, anthropometric and subjective measurements related to pain were performed for both groups, and blood samples were taken for laboratory analyses. Participants were provided with the appropriate study information before the enrolment and provided a written informed consent before the beginning of an intervention. The study was conducted between August 2020 and May 2022.

### Patients

The participants were recruited through social media. After the initial telephone interview, if no contraindications were present, the subjects were invited to the preliminary medical qualification carried out at the Department of Obesity Treatment, Metabolic Disorders and Clinical Dietetics of the Medical University of Poznań. Patients were enrolled on the basis of a complete medical history, a physical examination and additional tests, if available. Each patient who underwent the initial stage was referred for an MRI scan, the evaluation of the bone density and body composition. The final decision to admit patients to the clinical trial was made if there were no further contraindications for participation in the traction therapy.

The inclusion criteria for the study were: women aged 34–50 years with chronic lumbar pain lasting at least 6 months, stable body weight for the last month ± 2 kg, BMI 18.5–24.9 for normal-weight women, BMI > 30 for women with obesity.

The exclusion criteria were: menopause or pregnancy, secondary form of obesity, pain located elsewhere if stronger than the low back pain, pathologies and/or medications that might affect balance between pro and anti-inflammatory factors (e.g. inflammatory disease, rheumatoid arthritis, ankylosing spondylitis, systemic lupus, acute infection, cancer, overt inflammatory process of the respiratory tract, genitourinary system or within head and neck, alcohol abuse), type II diabetes, poorly controlled arterial hypertension, lipid disorders requiring pharmacological treatment in the last 3 months, chronic kidney disease, clinically significant impairment of liver function, acute coronary event, unstable angina, signs of heart failure, clinically significant arrhythmias or conduction disturbances, pacemaker implantation, neurological diseases including: vascular, posttraumatic, autoimmune, toxic and inflammatory in the last 6 months, previous surgery, post-accident mechanical injuries in the area of ​​the spine, medical diagnosis of spondylolisthesis, osteoporosis, history of syncope, uncontrolled mental illness, other conditions that might pose any risk to the patient during the intervention.

### Intervention

The examined patients underwent traction therapy based on introduction of traction forces stretching the spine, so that a number of beneficial functional and structural changes would occur, resulting in the reduction of pain. The traction table (Therapy Traction Couches and Packages, ST6567P, SEERSMEDICAL, Suffolk, UK) met the requirements of the European Union Directive 93/42/EEC). The application of traction forces lasted 30 min a day, 5 days a week, for 4 weeks (20 therapeutic sessions). A constant (continuous) traction mode was used, at the force level of 25% of the patient’s body weight during the first 5 treatment sessions, and gradually increased to 30% of the body weight.

## Mesurements

### Magnetic resonance imagining

In order to assess the degree of structural damage within intervertebral discs and adjacent anatomical structures, the patients underwent magnetic resonance imaging (MRI 1.5T, standard in 3 projections). All examined patients were assessed by the same experienced radiologist. The examination took place before the intervention.

All measurements listed below were made at two appointments: before therapy (pre) and after therapy (post).

### Dual energy X-ray absorptiometry

Body composition analysis was assessed using dual-energy X-ray absorptiometry (DXA, Lunar Prodigy device, GE Healthcare, Chicago, USA). Subjects were given complete instructions on the body composition analysis procedure and were instructed not to make any intense physical effort during 24 h prior to the measurement. All tests were performed in the morning. The total body fat mass and the lean body mass were determined using the standard scan mode (for normal-weight and moderately obese subjects) or the thick scan mode (for extremely obese subjects); the absorbed doses of radiation were 0.4 and 0.8 µGy, respectively.

### Visual analogue scale

The visual analogue scale (VAS) was used to measure participants’ back pain at the beginning and after therapy. A 10 cm VAS was used to evaluate the pain severity. The patients were asked to mark the score corresponding to their pain level in the last week on the pain scale, which was between 0 (no pain) and 10 (the worst pain imaginable). The scale was used in four categories: the maximum morning pain, the maximum night pain, the maximum pain while sitting, and the maximum pain while standing. The VAS score has already been used in patients with low back pain undergoing traction therapy [15] and appeared to be reliable in assessing pain severity [16].

### Pressure algometry

The pressure pain threshold (PPT) at the L1 level was assessed by the use of a digital force gauge (WAGNER FDIX Force One Digital Force Gauge, Greenwich, USA). Initially, each patient was introduced to the study and familiarized with the PPT testing. The PPT was described to patients as the threshold of first discomfort, so the PPT was the minimum pressure that caused a feeling of discomfort. Patients were examined in a lying forward position. Pressure, detected through the algometer sensor, was applied from above and perpendicular to the examined muscle. The PPT was measured bilaterally on the spinal erector muscle group, 2 cm laterally to the lumbar spinal processes L1. The test started at 0 kg/s and the pressure was increased at a rate of 1 kg/s. The participants were asked to say “now” when the sensation of discomfort became clear. All examinations were made by the same person from the research team. The average of three measures performed on each side was used for analysis. The algometer test is a reliable and reproducible method for detecting possible progress after interventions in patients with low back pain [17].

### Biochemical analysis

Blood samples for biochemical analyses were taken from a basilic vein, after overnight 12-hour fasting. The patients were asked not to take any anti-inflammatory drugs for at least 48 h prior to the blood sampling. In the serum samples, parameters were measured using commercially available enzyme-linked immunoassays. Aggrecan chondroitin sulfate 846 epitope was assessed using the test made by IBEX Pharmaceuticals Inc. (Canada). Adipsin and neuropeptide Y were analyzed using a Cloud-Clone Corp. (USA) ELISA kit. Leptin and GDF-15 were measured using tests made by BioVendor Research and Diagnostic Products (The Czech Republic).

### Statistical analysis

The data are given as means ± SDs (standard deviations). Baseline clinical characteristics were compared between groups using the Mann-Whitney U test, or the unpaired t-test if the data were normally distributed. The Wilcoxon rank-sum test or the paired t-test (for data with normal distribution) were used to analyze statistical significance of variables before and after the intervention. The Shapiro-Wilk test was used to check the normal distribution. A two-way repeated measures analysis of variance (ANOVA) was used to analyze the interaction of time × group in the case of GDF-15 concentration. The analysis of correlation between changes in anthropometric parameters and changes in biochemical parameters was carried out using the Spearman rank-correlation test, or the Pearson correlation test, if the data were with normal distribution. A sample size was determined on a basis of a pilot study on 23 chronic low back pain patients who received the same therapy, according to changes in the maximal morning low back pain. The power analysis indicated that a minimum 6 cases are necessary to yield at least 80% power of detecting an intervention effect as statistically significant at the 0.05 α level. The power analysis performed after the current experiment, confirmed that the sample size sufficient to yield at least 80% power of detecting an intervention effect as statistically significant at the 0.05 α level, with a detectable effect size of 0.8 was 10 cases in the normal-weight group and 6 in the obesity group. All calculations and statistics were performed using TIBCO Statistica 13.3 software.

## Results

**Fig. 1:**
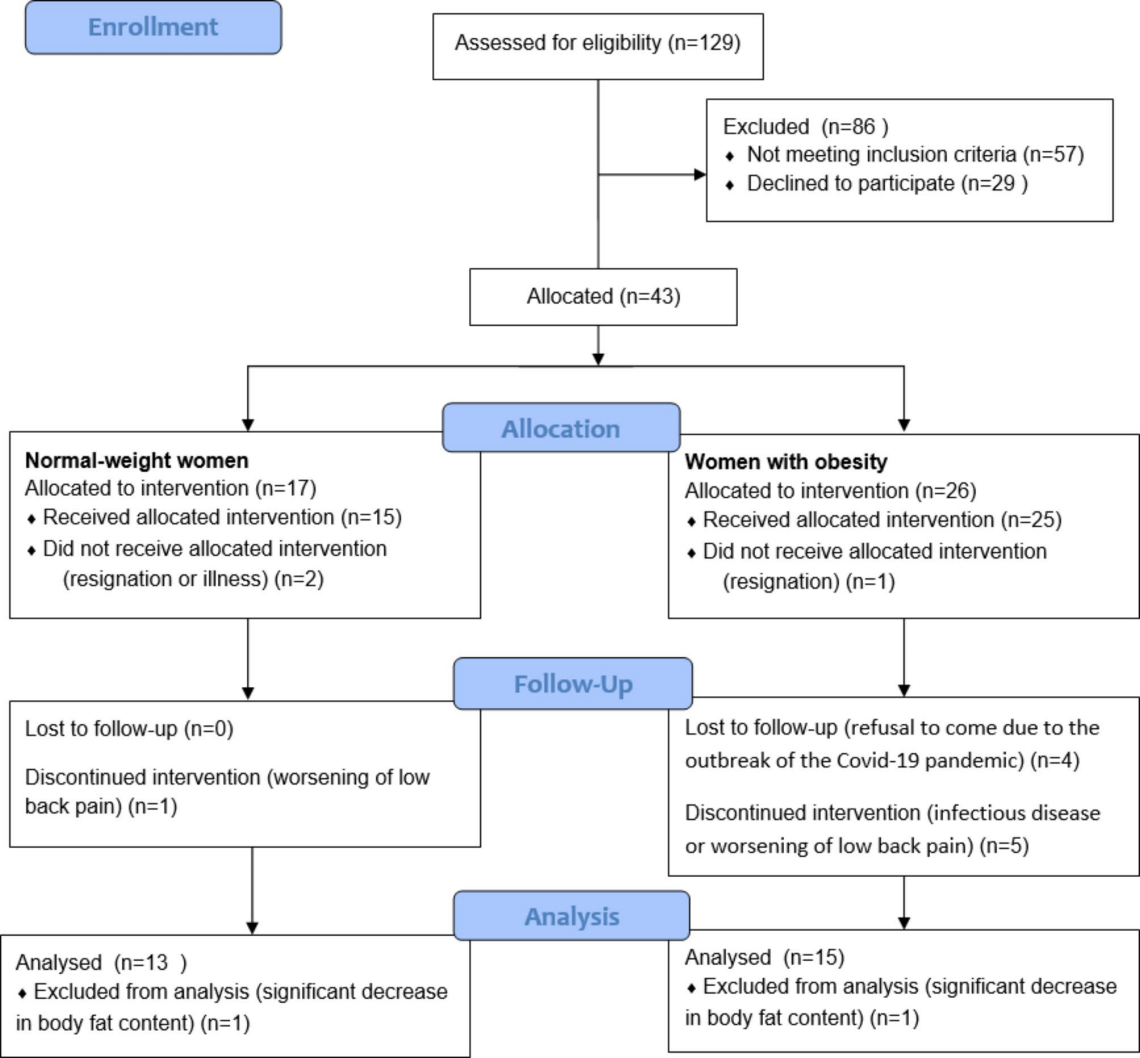
Schematic overview of the study timeline (the modified CONSORT flow diagram)

Figure 1 shows the overview of the study timeline, while Table 1 contains information about demographic and clinical variables of patients before the intervention. There was no statistically significant difference in the mean age between normal-weight women and obesity groups. According to the assumptions of the experiment, the patients differed in terms of the BMI and body composition.

The mean duration of low back pain was similar in both groups. Pre-intervention VAS values in both groups were similar for all four pain categories: morning, night, sitting, and standing. In both groups the highest mean intensity of pain occurred in the morning, up to 30 min after getting out of bed. On the other hand, night pain turned out to be the least intense symptom out of the four taken into account. When it comes to particular degenerations, a greater number of women among normal-weight group had protrusions, extrusions and spinal stenosis than among obesity group.

**Table 1:** Demographic and clinical characteristic of patients

	Normal-weight (n = 13)	Obesity (n = 15)	p value
**Age (years)**	42.7 ± 3.3	40.7 ± 5.7	0.2681^a^
**Body Mass Index (kg/m** ^**2**^ **)**	23.4 ± 1.0	35.2 ± 3.7	0.0000 ^a^
**Total Lean Mass (g)**	39.3 ± 3.6	49.9 ± 5.3	0.0000 ^a^
**Total Fat Content (%)**	35.0 ± 3.6	47.0 ± 4.6	0.0000 ^a^
**LBP duration (years)**	10.2 ± 7.0	9.3 ± 7.1	0.7511 ^a^
**Maximal morning LBP in the week before intervention (VAS 0–10)**	4.4 ± 3.2	4.0 ± 2.7	0.5963 ^a^
**Maximal night LBP in the week before intervention (VAS 0–10)**	2.1 ± 2.2	3.5 ± 3.3	0.2495 ^a^
**Maximal LBP at the sitting position in the week before intervention (VAS 0–10)**	3.9 ± 2.4	3.9 ± 2.8	0.9992 ^a^
**Maximal LBP at the standing position in the week before intervention (VAS 0–10)**	4.2 ± 2.4	3.7 ± 3.1	0.4896 ^a^
**Beck Depression Inventory**	4.8 ± 4.1	8.3 ± 6.8	0.1172 ^a^
	**Number of patients in the group**		
**Discopathy**	11	12	0.7496 ^b^
**Bulging**	5	6	0.9337 ^b^
**Protrusion**	10	6	0.0453 ^b^
**Extrusion**	4	0	0.0085 ^b^
**Multiple levels of hernia**			
**1**	2	6	0.1425 ^b^
**2**	7	4	0.1400 ^b^
**3**	1	0	0.2091 ^b^
**4**	1	0	0.2091 ^b^
**Annular disk tear**	7	4	0.1340 ^b^
**Spinal stenosis**	9	3	0.0074 ^b^
**Facet joint degeneration**	9	9	0.3882 ^b^
**Radiculopathy in MRI**	8	4	0.0606 ^b^
**Presence of radicular pain**	9	9	0.6103 ^b^

^a^U Mann-Whitney test or Student t-test, ^b^chi squared test

LBP, low back pain. VAS, visual analogue scale.

Table 2 shows the effects of traction therapy on the variables related to pain sensation of the lumbar spine in each group. The maximal low back pain decreased significantly after therapy in both groups of women, except the maximal night low back pain, which decreased only in the obesity group. The sensation of PPT in the lumbar spine did not change in both groups.

**Table 2:** Variables related to pain sensation of the lumbar spine measured before and after therapy

	Normal-weight (n = 13)			Obesity (n = 15)		
	PRE therapy	POST therapy	p value ^c^	PRE therapy	POST therapy	p value ^c^
**Maximal morning LBP** **(VAS 0–10)**	4.4 ± 3.2	1.5 ± 1.6	0.0128	4.0 ± 2.7	0.6 ± 0.7	0.0015
**Maximal night LBP** **(VAS 0–10)**	2.1 ± 2.2	1.0 ± 1.6	0.1141	3.5 ± 3.3	0.6 ± 1.0	0.0108
**Maximal LBP at sitting (VAS 0–10)**	3.9 ± 2.4	1.8 ± 2.0	0.0121	3.9 ± 2.8	1.2 ± 1.8	0.0043
**Maximal LBP at standing (VAS 0–10)**	4.2 ± 2.4	1.5 ± 1.7	0.0096	3.7 ± 3.1	0.8 ± 1.3	0.0026
**PPT- right side [kg/s]**	4.8 ± 1.4	4.1 ± 1.9	0.3455	3.65 ± 1.47	3.83 ± 1.68	0.6279
**PPT- left side [kg/s]**	4.9 ± 1.4	4.2 ± 2.1	0.2531	3.9 ± 1.4	3.9 ± 1.8	0.9850

^c^ the Wilcoxon rank-sum test or the paired t-test

Data are presented as the mean ± SD.

LBP, low back pain; VAS, visual analogue scale. PPT, pressure pain threshold.

**Fig. 2:**
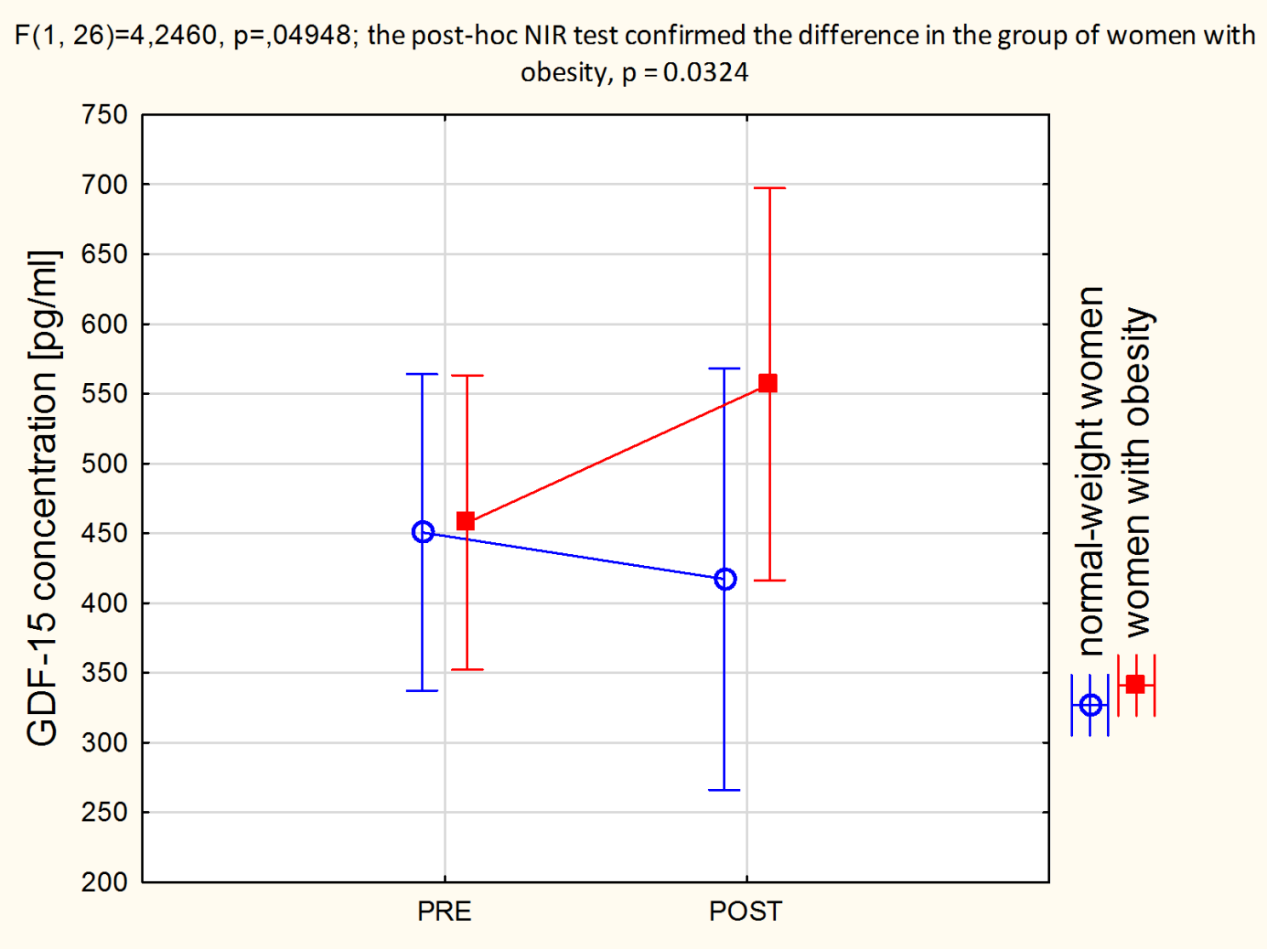
ANOVA interaction group x time: GDF-15 before and after therapy

Table 3 presents the effect of traction therapy on changes in the serum concentrations of biochemical substances. The mean concentrations of neuropeptide Y, leptin and adipsin did not change after therapy in both groups. For the obesity group a significant decrease in CS-846 concentration was found after therapy. GDF-15 was significantly reduced in the normal-weight group, while significantly increased in the obesity group. There was group vs. time interaction for GDF-15 concentration (Fig. 2).

**Table 3:** Low back pain potential systemic biomarkers measured before and after therapy

	Normal-weight (n = 13)			Obesity (n = 15)		
	PRE therapy	POST therapy	P value ^c^	PRE therapy	POST therapy	p value ^c^
**Leptin [ng/ml]**	14.0 ± 5.7	14.3 ± 9.5	0.9165	41.7 ± 10.0	42.3 ± 11.3	0.5701
**Adipsin/CFD [ng/ml]**	7.09 ± 1.87	7.25 ± 1.43	0.7535	8.02 ± 1.17	8.13 ± 1.00	0.6970
**GDF-15 [pg/ml]**	451 ± 190	417 ± 178	0.0392	458 ± 206	557 ± 321	0.0356
**CS-846 [ng/ml]**	20.1 ± 4.3	20.0 ± 2.7	0.8613	43.6 ± 83.9	42.1 ± 80.4	0.0356
**Neuropeptide Y [pg/ml]**	598 ± 144	580 ± 114	0.5953	865 ± 391	894 ± 454	0.6910

^c^ the Wilcoxon rank-sum test or the paired t-test

Data are presented as the mean ± SD.

CFD, Complemet Factor D, GDF-15, growth and differentiation factor 15. CS-846, aggrecan chondroitin sulfate 846 epitope.

**Fig. 3:**
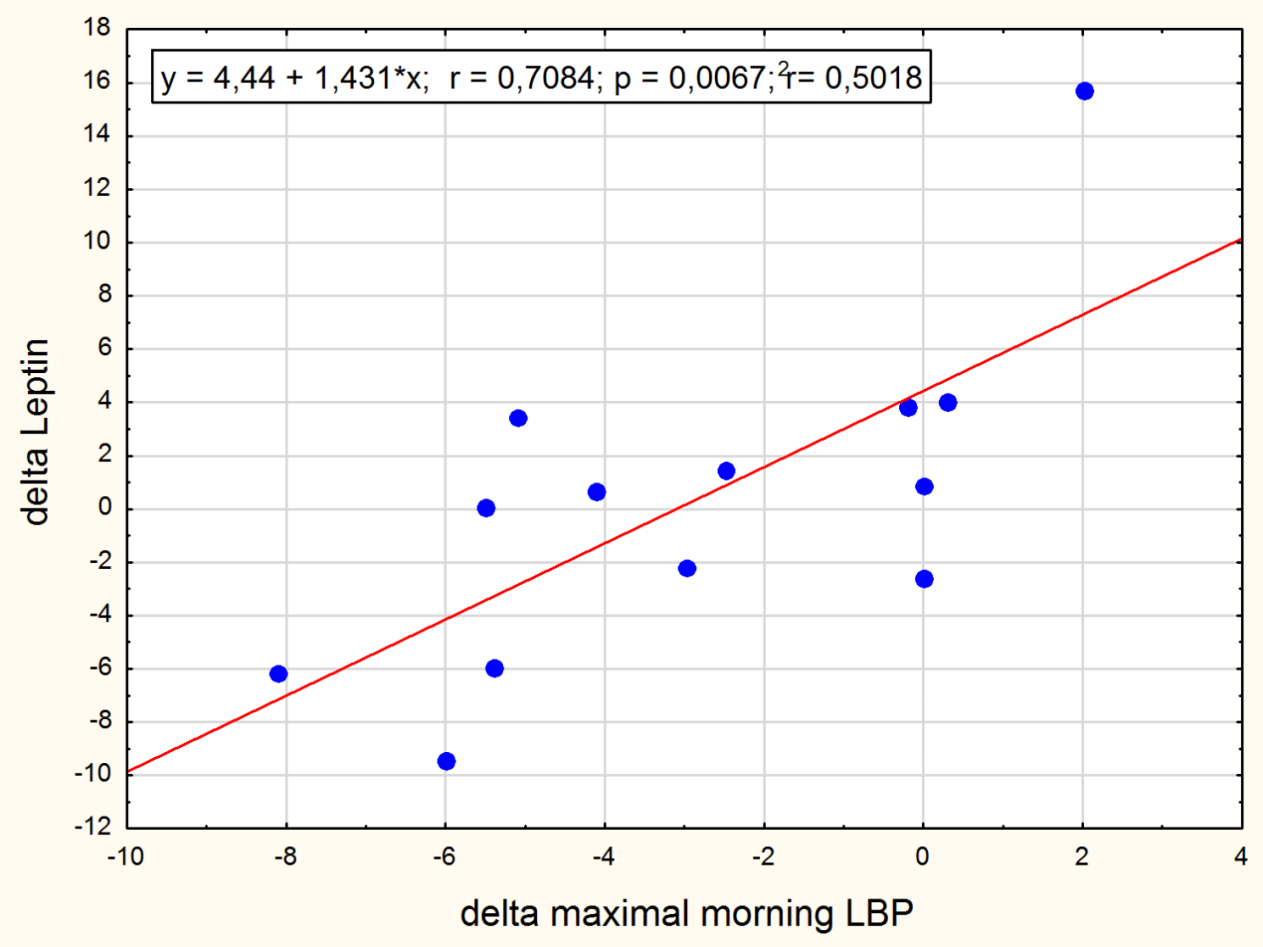
Pearson correlation between delta Leptin and delta morning LBP for normal-weight women

**Fig. 4:**
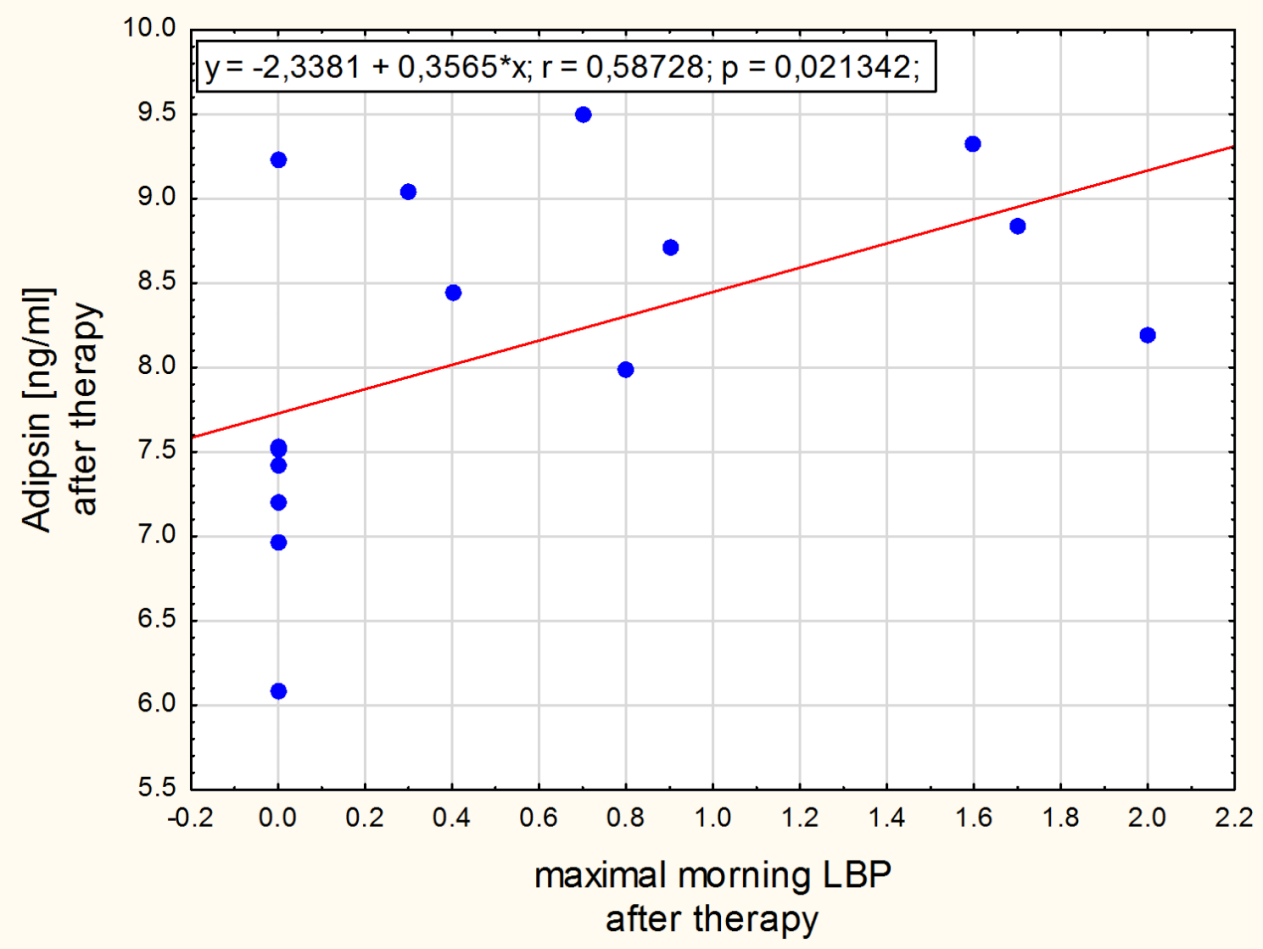
Spearman correlation between adipsin and the morning LBP after therapy for women with obesity

**Fig. 5:**
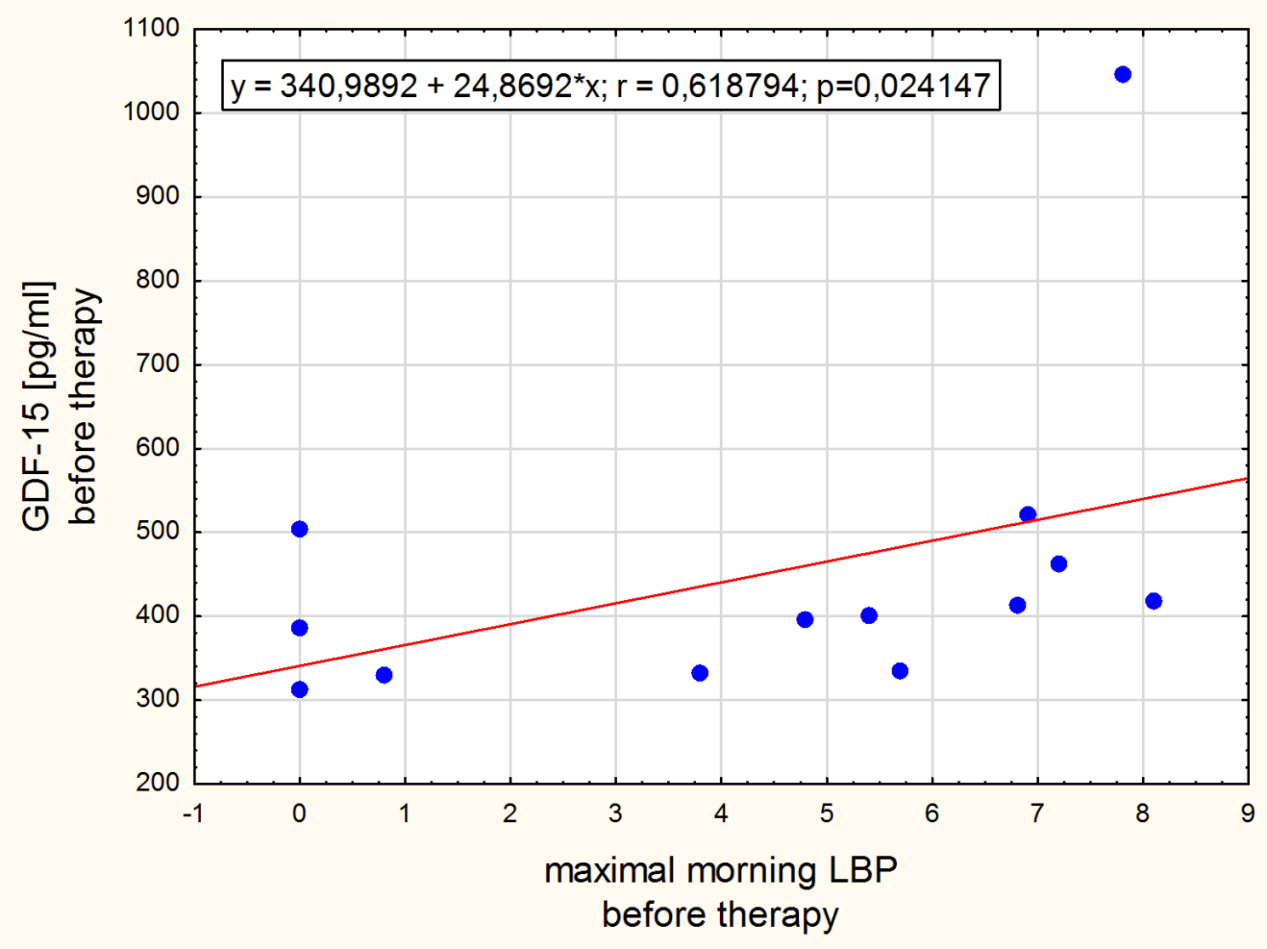
Spearman correlation between GDF-15 and the morning LBP before therapy for normal-weight women

**Fig. 6:**
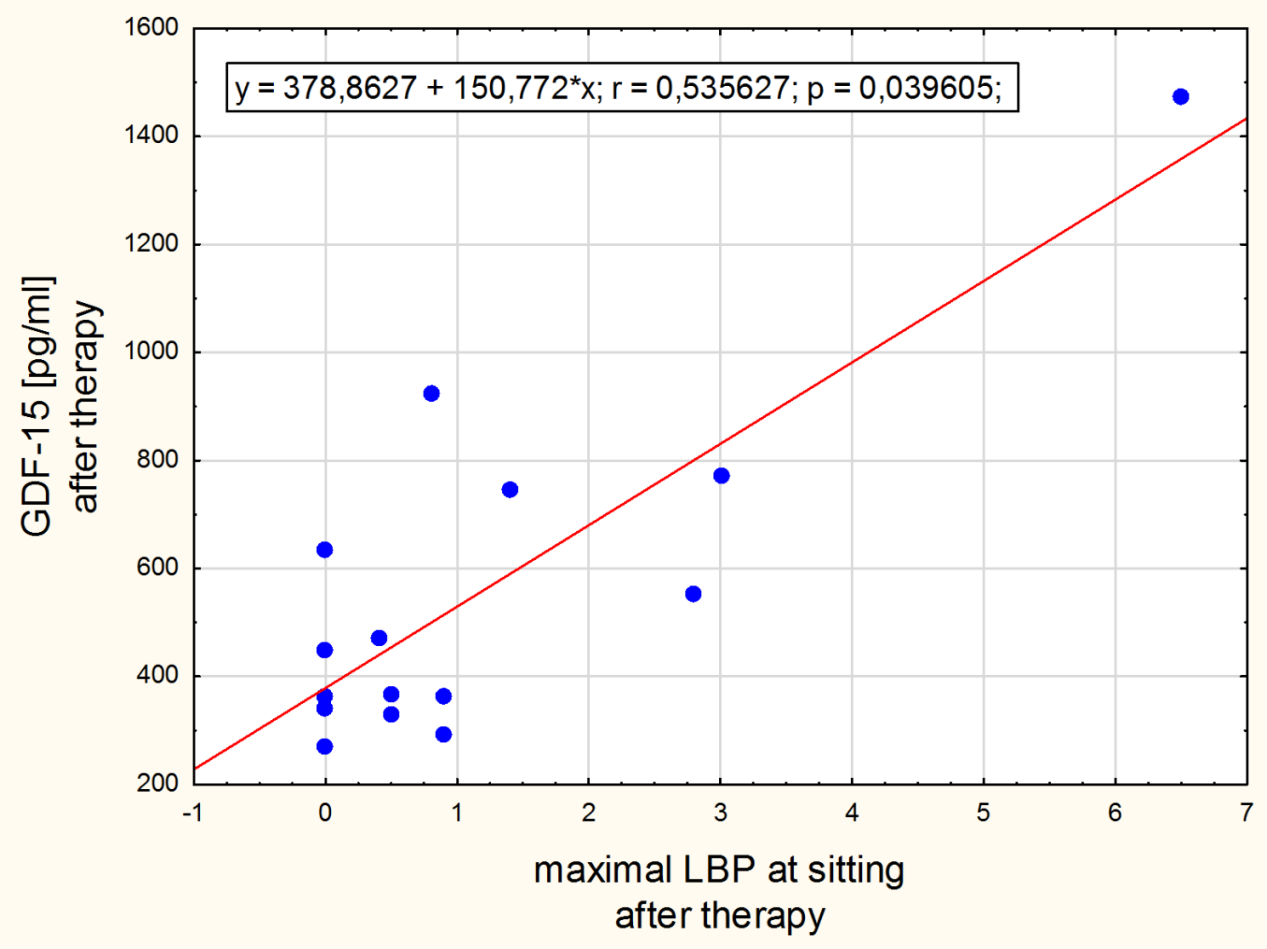
Spearman correlation between GDF-15 and the LBP at sitting after therapy for women with obesity

**Fig. 7:**
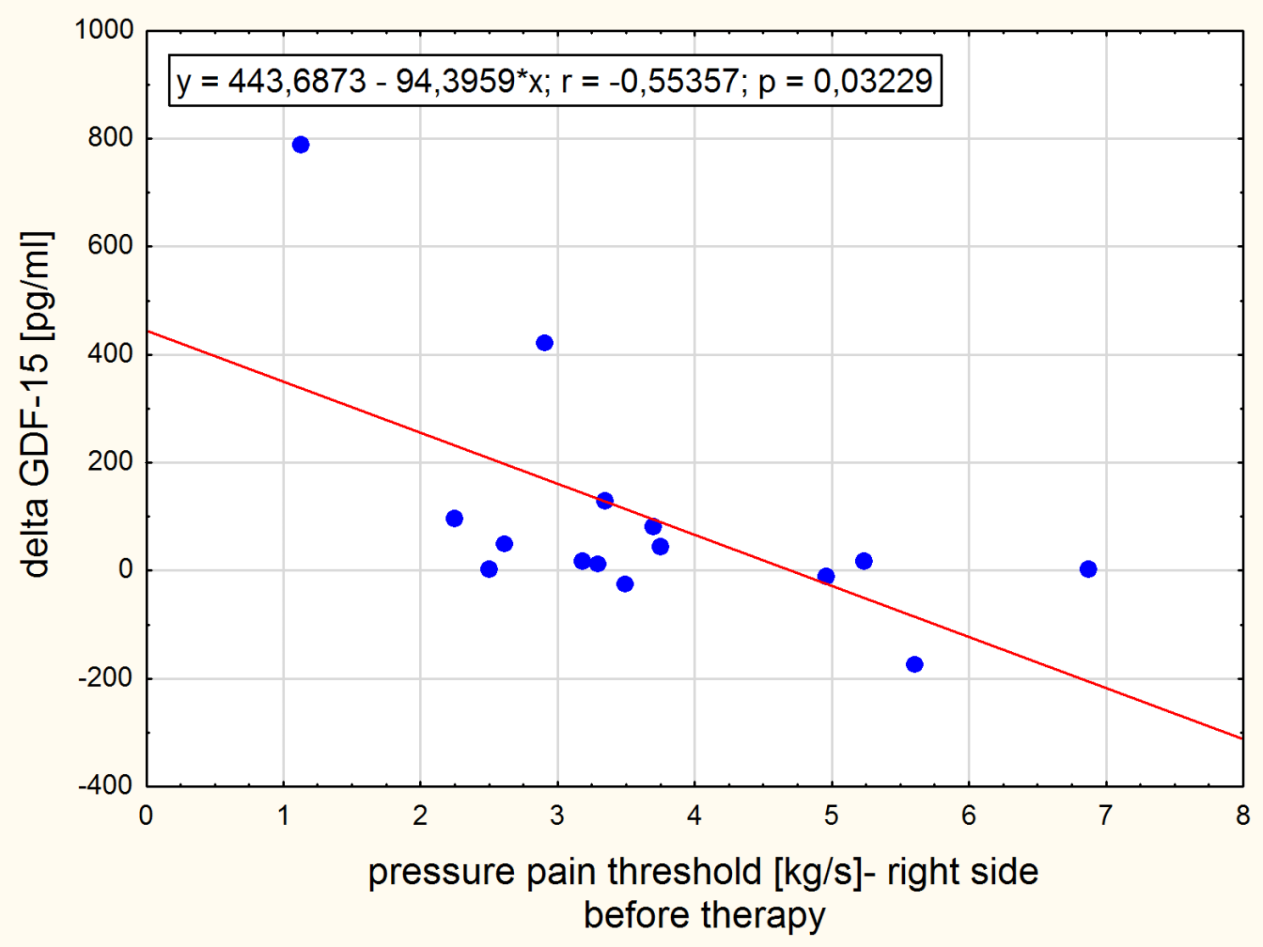
Spearman correlation between delta GDF-15 and PPT before therapy for women with obesity

The significant positive correlation was found between leptin concentration change and the morning low back pain change in the normal-weight group (Fig. 3). Intensity of the morning low back pain correlated with adipsin concentration after therapy in the obesity group (Fig. 4) and with GDF-15 concentration in the normal-weight group before the intervention (Fig. 5). After the therapy the correlation between intensity of low back pain while sitting and the GDF-15 concentration was found in women with obesity (Fig. 6). The PPT on the right side correlated negatively with a change in GDF-15 concentration in women with obesity before therapy (Fig. 7).

## Discussion

Research on the assessment of biochemical indicators in patients with chronic low back pain contributes to a better understanding of the mechanisms of pain formation in the spine. Identification of specific biomarkers of the chronic low back pain would improve standards of diagnosis and would help monitoring of the effects of therapy. In this study, we assessed the effectiveness of the common therapeutic method, i.e. the lumbar traction, and we showed that traction therapy significantly reduced the intensity of maximal low back pain in the morning, at sitting, and at standing, both in women with normal body weight and with obesity. Moreover, the traction therapy resulted in a change in the concentrations of two out of five tested biochemical substances (CS-846 and GDF-15) which therefore aspire to be the low back pain biomarkers in obese women. We also demonstrated relationships of GDF-15, leptin and adipsin concentrations with the perception of pain, in particular with the morning pain, which is considered a characteristic symptom of inflammatory back pain.

### Subjective assessment of therapeutic effects of traction

There were many randomized controlled trials which indicated that traction might be an effective intervention in the treatment of patients with low back pain, resulting in a significant reduction in pain intensity after segmental traction therapy [15, 18, 19]. It was also reported that patients with hernia of the lumbar disc experienced considerable pain relief after continuous lumbar extension [20]. A significant reduction in the intensity of all types of pain (morning, night, at sitting, at standing) determined by the VAS scale was noted in our study as well, with the more noticeable effect in obese women. For women in the normal-weight group, the exception was the night pain (see Table 2), probably due to its relatively low intensity before the therapy.

Long-term chronic pain symptoms can influence the central processing mechanisms at the neurophysiological level, thus changing subjective pain perception [21]. It is believed that central pain sensitization can be measured distally using the PPT method [22]. Changes in PPT and tissue hyperalgesia in patients with chronic low back pain were evaluated by Zicarelli, et al. (2021) [17], who concluded that the reliability of PPT in acute patients is high, but its usefulness in chronic conditions remains uncertain due to the wide variability of methods used in therapy. Results of a recent study by Goode et al. (2022) [23] indicated that PPT is associated with structural degenerative changes and self-reported pain. The authors found lower PPT values, ​​indicating higher pain sensitivity, in people diagnosed with low back pain compared to people with structural changes in the spine, but without pain. Low PPT values ​​in patients diagnosed with low back pain may be explained by the fact that injured tissues release inflammatory mediators, which excite nociceptors in the local area, maintaining the sensory excitation and nociceptive hypersensitivity in the injured region of the body [24]. Therefore, one can assume that PPT may have prognostic potential for differentiating phenotypes of lumbar spine degeneration and low back pain.

Nevertheless, changes in PPT were observed after the use of various physiotherapeutic methods in the treatment of the chronic low back pain [25, 26]. This study was however the first attempt to measure PPT changes ​​after traction therapy. We showed that the mean values of PPT for both normal-weight and obese women were within the range of values previously reported in the literature [23, 27], but no changes were observed ​​after 20 sessions of traction therapy. Additional studies are still needed to evaluate whether PPT may be a useful pain measure to differentiate individuals who might have structural findings, but it is likely that only therapies that have long-term effects and are aimed at reducing inflammation would improve this parameter.

### Biomarkers of low back pain

A biomarker should objectively reflect physiological and pathological biological processes or the response to a therapeutic intervention [28]. Since the active inflammatory process seems to be the overriding factor in the pathogenesis of degenerative changes in the intervertebral discs, factors related to inflammation pretend to be biomarkers of damage to the structures of the spine. Especially factors related to the metabolic activity of adipose tissue should be considered, as the excess of adipose tissue creates a local pro-inflammatory status. In the case of back pain there is also debate about the effects of non-inflammatory substances, such as cartilage metabolism markers such as CS-846 or neuropeptide Y.

#### Leptin and adipsin

The relationship between back pain and obesity is a frequent subject of research. In a recent study, the prevalence of high intensity back pain was significantly associated with body fat content, particularly in the android region, and with high android to gynoid fat ratio [29]. This indicates that in the pathogenesis of back pain the mechanical load with excessive body weight is less important than the specific properties of adipose tissue. Abdominal adipose tissue is highly metabolically active, which is manifested by the secretion of adipokines and inflammatory cytokines, thus maintaining chronic low grade inflammation [30].

Leptin and adipsin, responsible for maintaining body weight and controlling appetite, are considered to be particularly related to the pathogenesis of back pain through the metabolic pathway. Both adipokines exhibit pro-inflammatory [31] and nociceptive [32] properties. It is suspected that high leptin concentration is associated with the reorganization of the atherosclerotic cytoskeleton [33], and its role in the degeneration of intervertebral discs has been so far confirmed in vitro [34]. However, in studies conducted in overweight and obese people, adipsin and leptin concentrations were higher in those with back pain. Adipsin, unlike leptin, remained associated with back pain also after adjusting for BMI, waist circumference and fat mass [13].

In our study, we assumed that if leptin or adipisin concentrations changed under the influence of traction forces, the study model with two experimental groups would allow us to determine whether the relationship of substances with back pain applies only to obese or also to slim women. Although the concentrations of leptin and adipsin in the subjects were clearly higher in obese women, their levels did not change immediately after the applied therapy (see Table 3). Interestingly, changes in leptin concentrations strongly correlated with changes in the intensity of morning pain experienced in the normal-weight group: the more leptin levels decreased, the more the intensity of morning back pain decreased (see Fig. 3). No such observation in obese women might result from other causes of increased leptin levels in this group.

Adipsin concentration correlated with the intensity of the morning low back pain after the therapy only for women with obesity (see Fig. 4), which is consistent with the study of Brady at al. [29]. It is possible that in people with excessive body fat, other phenotypes of back pain predominate and that inflammation originating from adipose tissue becomes more important in its pathogenesis, in contrast to lean people, in whom structural changes predominate spontaneously exacerbating inflammation. Other authors linked both morning and night back pain to the occurrence of inflammation in the spinal tissues [35, 36]. Whether adipsin is a compound that correlates only with back pain which is associated with adipose tissue inflammation remains an open question.

If the above assumptions were true, therapy of back pain in obese people should be primarily aimed at reducing body fat. Adipsin, also known as Complement Factor D, has catabolic rate of plasma reaching 60% per hour, which is associated with its low concentrations in non-pathological situations [37]. On the contrary, its high concentrations reflect pathological processes in adipose tissue. Therefore, the concentration of adipsin, a complement component responsible for activating the inflammatory response [38], may indicate the source of inflammation contributing to back pain and this way suggest the most effective therapy to restore the patient’s functional fitness and well-being. However this important issue requires a different design of the research.

#### GDF-15

Adipose tissue dysregulation and sarcopenia or sarcopenic obesity are processes related to each other and underlying back pain. The intracellular concentration of lipids in multifidus muscles was observed using MR spectroscopy and their content was confirmed to correlate with low back pain [39]. The growth and differentiation factor-15 is a hypothetical factor responsible for stimulating above mentioned processes, and thus low back pain. Independently of age, gender and body composition higher concentrations of GDF-15 were observed in people receiving medical consultations and in people with more severe disability related to low back pain than in people with a lower degree of disability [4]. The exact mechanisms of GDF-15 secretion and its usefullness are not yet known. Its concentrations rise during cellular stress, e.g. in response to tissue damage, in chronic inflammatory diseases, but also due to metformin treatment and exercise [40]. It is presumed that high GDF-15 level reflects a physiological response aimed at restoring metabolic homeostasis.

In our study, the concentrations of the GDF-15 cytokine were in the range of physiological values ​​[40], but after traction therapy its levels decreased in the normal-weight group of women, while increased in women with obesity. Traction therapy, like physical exercise, is an intervention mechanically interfering with the tissues. Considering that GDF-15 is a stress-responsive factor regulating inflammatory pathways, its decreased status in the normal-weight group of women suggests better effectiveness of repair processes. The inflamed tissues could heal after 4 weeks of traction therapy. The increased concentration of GDF-15 in the group of obese women may indicate just the initial stage of positive repair changes. It is also likely that in obese women the GDF15 signaling pathways that regulate homeostasis are malfunctioning or less efficient, or there are more active sources of GDF-15. Adipose tissue and immune cells entering the liver and adipose tissue in the process of developing obesity may abundantly express the GDF-15 [40, 41]. Another element in the puzzle confirming that GDF-15 concentrations reflect the intensity of the symptoms associated with chronic back pain syndrome are the relationships observed in this clinical trial (see Figs. 5, 6 and 7), of which the latter is particularly interesting as it suggests that the lower the sensitivity threshold in the lumbar region (indicating a greater problem), the stronger the body’s response to GDF-15 secretion following traction therapy.

#### CS-846

Degeneration of the intervertebral discs, the cartilage end plate of the vertebral bodies and the intervertebral joints seems to be a major direct cause of lower back pain [42]. One of the hallmarks of cartilage degenerative changes is the loss of key components of the extracellular matrix, which is manifested by an increase in its matrix turnover, i.e. increased production of building components [43]. The important building components of cartilage, responsible for its proper hydration, are proteoglycans, made of aggrecans. Degradation of aggrecans lead to dehydration within the intervertebral discs, which significantly reduces their ability to withstand loads [1]. In this study we determined the concentration of a cartilage proteoglycan aggrecan turnover epitope CS-846, recognized as the marker of aggrecan turnover in cartilage tissue, which is present only on newly synthesized chondroitin sulfate aggrecan molecules [44]. When cartilage degradation progresses, the CS-846 epitope begins to lose its matrix, which automatically contributes to the occurrence of its higher concentrations in the blood. Its high concentrations in the serum may therefore indicate ongoing anabolic processes, especially in the early stage of degeneration development [45].

Therefore, a significant reduction of CS-846 epitope observed after traction therapy in the group of obese women (see Table 3) may indicate beneficial changes, e.g. slowing down the degradation of cartilage resulting from the decompression of the perivertebral structures. It was shown in the study of Schaaf et al. (2021) [46] that patients suffering from low back pain and responding to epidural steroid injections with a reduction of pain had lower levels of CS-846 at baseline. Therefore it is possible that the prognostic value of CS-846 might be extended to monitoring long-term therapies aimed at slowing down the degeneration of the lumbar spine structures or the levels of CS-846 would be useful to discriminate back pain by etiology. However, the above conclusions should be treated with caution because the tissue source of this epitope cannot be determined using peripheral samples.

#### Neuropeptide Y

One of the recently recognized properties of neuropeptide Y is pain modulation. More and more scientific evidence confirms that this regulation applies to nociceptive transmission to the spinal dorsal horn from the periphery [47]. In experimental studies on rabbit cells taken from the annulus fibrosus of the intervertebral discs, increased expression of the compound in the cells was found after administration of an inflammatory substance [48]. An increase of neuropeptide Y plasma level was also observed in rats with chemically induced inflammatory low back pain, immediately following a therapeutic procedure involving manipulation of damaged soft tissues [49].

Studies in humans also confirm the relationship of neuropeptide Y with back pain. Its blood concentrations correlated strongly with pain and pain-related function in elderly people with axial low back pain [3]. On the other hand, The Johnston County Osteoarthritis Project with 731 patients did not show a direct relationship between neuropeptide Y concentration and low back pain. However, after dividing the patients into experimental phenotypes, lower concentration of neuropeptide Y was found in the subgroup with the highest number of patients with low back pain and a low pain threshold [23]. Moreover, in the aforementioned study examining effects of epidural steroid injection [46], respondents to the steroid treatment had higher neuropeptide Y concentrations. The authors suggested that biomarkers that provide information on the subjects’ overall pain experience, are more relevant to predict response to a procedure which targets pain than markers aimed at detecting ongoing reconstruction processes in an unknown place [46].

Our study, however, did not confirm changes in the concentration of neuropeptide Y under the influence of traction forces, neither in the normal-weight group, nor in the group of women with obesity. This indicates that additional research is needed to dispel doubts about the potential value of neuropeptide Y as a biomarker of low back pain.

### Study limitations

The low size of the groups of patients is a weak point of this study. On the other hand, the groups are very homogeneous, ensuring the elimination of many factors that interfere with research on systemic biomarkers, such as age, presence of menopause, body weight or its composition. The value of the project would also increase by the appointment of additional control measurements of the concentrations of tested compounds, which appeared clear after completing the study. At least one of such points could be assigned during the therapy, e.g. after 2 weeks of its duration. Moreover, control measurements one week, two weeks or one month after the end of therapy would possibly reveal the point at which tissues achieve the state closest to homeostasis.

## Conclusion

The comparison between the groups of women with normal body weight and women with obesity suggests different origin of back pain in both groups. It is likely that excessive body fat, which aggravates the inflammatory processes is more often an important factor in the development of low back pain in obese people. At this stage of the research, the tested biochemical substances cannot be considered as the low back pain biomarkers useful in the assessment of the effectiveness of traction therapy, which suggests the need for further research in this direction. The obtained results, however, shed light on the possibility of using biochemical substances in determining the etiology of low back pain as well as in the prognosis and monitoring of its treatment.

## Data Availability

The datasets used and/or analysed during the current study are available from the corresponding author on reasonable request.

## Abbreviations

BMI: body mass index
VAS: visual analogue scale
PPT: pressure pain threshold
CS-846: aggrecan chondroitin sulfate 846 epitope
GDF-15: growth and differentiation factor 15
LBP: low back pain
